# Report on the Progress of Practical Medicine, Pathology, and Therapeutics

**Published:** 1845-11

**Authors:** W. H. Ranking


### RECORD OF MEDICAL SCIENCE.

### *Report on the Progress of Practical Medicine, Pathology, and Therapeutics.*

BY W. H. RANKING, M. D.

### § I.—*Zymotic Diseases.*

1. *Continued Fever.* The period which our present Report professes to embrace has not been remarkable either for the number or importance of the communications upon the subject of fever. We may, however, observe that Dr. Davidson has presented us with a series of aphorisms on the treatment of typhus which cannot fail to recommend themselves to notice by their simplicity, and perfect accordance with those enlightened views of the pathology of the disease which are prevalent in the present day. These observations will be found among our abstracts, and therefore need not be further insisted upon. On the same subject we may refer to memoirs by M. Sandrat and M. Delaroque, in each of which the treatment is discussed at considerable length. The former relies entirely upon the repeated exhibition of Seidlitz water; M. Delaroque endeavours to point out the beneficial effects of more energetic purging. This latter memoir, in other respects, contains nothing which is either new or interesting to the British practitioner, and appears, in fact, to be a mere repetition of the principal points in a work published by the author in 1839, and of which as much notice as it deserves will be found in the *British and Foreign Medical Review*, Oct. 1844.

In a discussion upon the pathology of typhoid fever, in which the Académie Royale de Médecine Belge has lately been engaged, much difference of opinion existed upon the principal propositions contained in a paper read by M. Mascart. The author, in acknowledging the origin of fever from miasmatic intoxication, endeavours to show:—1st, That the effect of the poison is to diminish the quantity of fibrin in the blood; 2dly, That the blood thus diseased excites an inflammation of the lining membrane of the blood-vessels, which inflammation is to be considered as the natural means of restoring the deficiency of fibrin, according to the laws established by Andral and Gavarret; and 3dly, That the ulceration of the intestinal follicles is likewise a natural process by which the morbid poison is eliminated, and consequently that it should not be interfered with. These opinions were combated seriatim by several of the members present, and by M. Fallot in particular, who clearly exhibited their untenability. The first proposition, as he observes, is negatived by the researches of Andral and Gavarret, who distinctly state that the blood undergoes little or no alteration in typhus until the latter stages of the disease. It is also, we may remark, opposed by the more recent investigations of MM. Becquerel and Rodier, which tend to the belief that the fibrin is not diminished in typhoid fever at all, excepting in its most adynamic type, or when bloodletting has formed a part of the treatment. The second proposition is met by the serious objection that if diminution of fibrin does in reality excite vascular inflammation, that phenomenon ought to occur in all cases in which this constituent of the blood is notably deficient. Such, however is not observed to be the fact either in scurvy, or in the cases in which the blood has been artificially defibrinized, as in the experiments of Magendie. The third proposition, namely, that the inflammation and ulceration of the intestines is a salutary process, is scarcely worthy of formal refutation, the smallest experience being sufficient to show that this lesion forms, in all cases in which it is present, the most serious obstacle to recovery, and that the diarrhœa which it produces is of all the symptoms the one most difficult to subdue.

2. *Remittent Fever* has been treated by Dr. Swett in a clearly written essay, the principal object of which is to confirm the opinion previously advanced by Dr. Steward, that the most characteristic lesion in the severe remittents of warm climates is a peculiar condition of the liver, which consists in a blueish gray or slate colour of the parenchyma, with the interspersion here and there of patches of a bronzy tint. The granular structure is at the same time quite distinct, each granule being surrounded by a ring of vascular injection.

3. *Scarlatina.* Under this head we have to mention a paper by Dr. Alison, calling the attention of the professsion to the not uncommon occurrence of pericarditis as a complication of the cutaneous affection. The author cites the names of those writers who do, as well as of those who do not, allude to this subject, and it appears that the latter are greatly in the majority. From this fact alone we should be disposed to think that the frequency of the complication has been somewhat exaggerated. That the pericardium, however, is liable to inflammation in the course of scarlet fever may be seen in the writings of Drs. Joy and Burrows as mentioned by the author, in those of Dr. Golding Bird, and likewise in the *Stuttgard Collection of Children's Diseases*, where the occurrence of purulent collections in the pericardium during scarlet fever is alluded to by Von Ammon. There does not appear to be any peculiarity in the type of the fever in which this complication exists, neither does it require any special modification in the treatment which is best adapted to the idiopathic disease; it is necessary merely to bear in mind, that as it supervenes upon an affection which depends upon the imbibition of an animal poison, great circumspection is necessary in the employment of depletory measures.

Dr. Wilshire combats the opinion that the dropsy which follows scarlet fever is in general an inflammatory disease, and maintains, on the contrary, that it is rather one of an asthenic character. In this view of the case we entirely concur, believing it to be especially true as regards the children of the poor, who from circumstances, the operation of which is sufficiently evident, are particularly exposed to the sequelæ of eruptive diseases. Dr. Wilshire speaks highly of the iodide of potassium in a bitter infusion, as a remedy in these cases.

Dr. Golding Bird, who took part in the discussion which was elicited by the enunciation of the foregoing opinion, speaks of two forms of scarlatinous dropsy, one simple and manageable, the other highly dangerous, inasmuch as the blood contains some of the uneliminated elements of the urine. In another place he endeavours to explain the more frequent supervention of the dropsical symptoms upon the slight than the severe forms of the eruption, somewhat after the following manner. He supposes it to be conceded that the disease originates in a poison, the primary location of which is in the blood. The two principal features of the disease, namely the eruption on the skin, and the erythismic excitement of the mucous surfaces, are the means which nature makes subservient to the elimination of this poison. If, therefore, the rash be well developed, the system is effectually disembarrassed of the morbific agent; but if not, the poison not being all excreted, some of its recognized after-effects result. How, he inquires, are these effects induced? Granting the existence of the imperfectly exhausted materies morbi in the blood, attempts will be made after the subsidence of the eruption, to eliminate this matter by one or other of the emunctories of the body. The kidneys are the organs by which matters in solution in the blood are usually excreted; and either from deficient determination to the skin in the first instance, or from the application of cold subsequently, are made to assume a supplementary duty; their capillaries therefrom become dilated, and congestion ensues. The consequence of this is a double lesion of their function; on the one hand, exudation of the albuminous element of the blood, and on the other retention of the nitrogenized products. Contemporaneously with this, serous effusion generally, but not invariably occurs.

Dr. Corrigan describes a peculiar and very fatal form of scarlatina anginosa, which he states has not been noticed by any preceding writer. In this, however, he is in error, as the same form is clearly alluded to by Dr. Watson (Lectures, vol. ii. p. 758) who, it may be observed, also acknowledges its great severity. This variety of the disease consists in the rapid swelling of the parts beneath the angle of the jaw, without any corresponding inflammation of the throat internally. The swelling occasionally arrives at an enormous size, and the child usually sinks under the sloughing of the cellular tissue, or from the effects of pressure upon the cervical blood-vessels. Dr. Corrigan especially warns the surgeon against opening the tumour, or making incisions through the integuments, which he might otherwise be tempted to do, under the impression that he had met with a case of diffused cellular suppuration. He has never seen the slightest benefit from such a proceeding, and when it has been practised the cellular tissue, instead of containing pus, has been found to be infiltrated with dirty-looking serous fluid. The only treatment which can be relied upon is that of the preventive kind, and which consists in the application of relays of a small numbe of leeches; but this must be instituted before the inflammation is fairly established, or the patient will, according to Dr. Corrigan, be inevitably lost. The same affection is apparently referred to by M. Rostan as forming a serious complication in typhoid fever.

In connexion with the subject of scarlatinal dropsy, Dr. Golding Bird details a simple method of detecting urea in the blood and serous fluids. The blood having been allowed to coagulate, the serum is to be decanted, and shaken up with an equal quantity of rectified spirits, which cause the precipitation of albumen. The mixture is then to be filtered, and the filtered fluid, after having been evaporated to two drachms, is to be treated with an equal bulk of dilute nitric acid, and again filtered. The filtered fluid being slowly evaporated on a watch-glass, will exhibit the feathery crystals of nitrate of urea. The treatment recommended by Dr. Bird in this form of dropsy is simple, and, according to our experience, generally successful. It consists in the endeavour to promote active diaphoresis by the use of the warm-bath and small doses of the Vin. ant. potass. tart. in julep. ammonia.

4. *Glanders.* The possibility of the transmission of this fatal disease from animals to man, though long doubted, and even by some disbelieved at the present time, is nevertheless a fact, which may be considered as incontrovertibly established. The case reported by M. Pavard is a faithful portrait. To those of our readers who are anxious to be acquainted with all that is known upon the subject, we would suggest the perusal of a paper by M. Rayer, published in 1837, of which an excellent notice will be found in the *British and Foreign Medical Review*, January, 1842.

### § II.—*Diseases of the Nervous System.*

5. *Insanity.* The melancholy subject of mental derangement receives increasing attention. Among the most important additions to our knowledge in this department, which the last six months have produced, we may refer to the Reports of Dr. Conolly, on the Lunatic Asylums of Paris and other French towns, and to that of the Commissioners upon the condition of Lunatic Asylums in England and Wales. The latter affords a sad picture of the abodes of pauper lunatics in particular in this country, but in some degree also inculpates those intended for the reception of the wealthier classes. There is one practical fact elicited by the inquiry of the commissioners which, although not new, is yet worthy of repeated mention ; it is that of a large proportion of cures among patients admitted within three months of their first aberration, as compared with those who have been kept at home, or in the workhouse for longer periods.

In a series of lectures now in the course of publication by M. Baillarger, the hereditary nature of insanity is strongly insisted upon. According to the observations of M. Baillarger, the transmission of the malady is to be apprehended under any of the following circumstances :—when the father or mother is insane at the time of or previously to conception ;—when the father or mother have insane blood relations;—when they are remarkable for eccentricity or violence of character:—when they have suffered from diseases of the nervous system; if either have committed suicide, or has been addicted to drinking, or is old relatively to the other.

Mr. Grantham and Dr. Winslow remind us of the great importance of paying attention to the premonitory signs of mental derangement, for it is at this time that the patient stands as it were upon neutral ground, and that the question of his restoration is often to be decided. The symptoms indicative of the approach of mania which are clearly described by the latter writer, are, he thinks, in most cases to be referred to bodily derangement, and do not depend, as is thought by some, upon purely psychological disturbance.

Some useful remarks upon that form of insanity which is occasionally seen to follow the exhaustion induced by prolonged suckling, are to be found in Dr. Ashwell's late work. The treatment therein recommended is such as would be instituted by any well informed practitioner, and embraces weaning the child as a sine qua non, together with the persevering use of tonics, combined with sedatives, pure air, and exercise proportioned to the strength.

6. *Apoplexy.* The pathological conditions of the cerebral portion of the nervous system, have always been held to be the most difficult to be comprehended of any to which the human frame is amenable. There is a want of correspondence between lesions and symptoms, a want of uniformity in the manifestations of diseased action, which renders the subject one of surpassing intricacy. Not the least remarkable property connected with cerebral diseases, is the fact which is frequently manifested in the malady under consideration, that precisely the same train of symptoms may be produced by causes diametrically opposite; as, for example, by too much blood circulating in the vessels of the brain, and too little,—by a state of plethora as well as by that of anemia. This being the case, it becomes a question of the most vital consequence in practice to determine, to which of these conditions a given apoplectic seizure is to be attributed. This question is not so ready of solution as might at first sight be supposed. The tendency of medical men in general is too much to regard all apoplectic attacks as the result of fulness, whence bleeding is as much too frequently adopted. But that such a mode of viewing the phenomena of the disease as a general rule is as erroneous as the treatment arising out of it is dangerous or even fatal, will, we are convinced, become daily more universally admitted. The small amount of influence which the practice of bloodletting exercises in the cure of apoplexy is sought to be established in a late publication by Mr. Copeman, upon the evidence of statistical data. Although we cannot admit that all the cases upon which the deductions of this author are based, are the best which might have been selected, they are still, we think, sufficiently trustworthy to be recorded as an approximation to the truth. It appears that of 155 cases of apoplexy in which the treatment is specified, 129 were bled, and only 26 were not; of the former number, 51 recovered, and 78 died: the cures, therefore, were as 1 : 1½ ; the deaths, as 1 : 1 2-3ds. Of the number not bled, 18 were cured and 8 died, the proportion of cures being as 1 : 1½ ; of deaths, as 1 : 3¼. Abstracting a certain number of these cases in which the bleeding consisted in the application of a few leeches only, we reduce the figures to 112 ; of those 38 recovered, and 74 died, i. e., there were two deaths where bleeding was practised to one cure. Although, as has before been said, these facts are to a certain degree imperfect, and like all deductions connected with so inexact a science as that of medicine, which are based upon the numerical method, are open to objection, they must, nevertheless, have the tendency as far as they go to place the abstraction of blood as a general remedy in apoplexy in an unfavourable light.

It is still however unquestionable, that in a certain number of cases of apoplexy, free bloodletting is imperatively called for. How then are these cases to be distinguished ? The author above-mentioned places great confidence in the appearances of distension of the external vessels of the head ; but this rule will in many cases be fallacious, in those patients in particular in whom constant exposure to the vicissitudes of temperature and season has produced a dilated condition of the capillary vessels of the face. In these persons a florid countenance is perfectly compatible with a state of system intolerant of the loss of blood, and would, therefore, if relied upon, lead to serious errors in practice.

A surer guide in a doubtful case will be found in some observations by Dr. Marshall Hall, which we have considered of sufficient value to merit a place in our Abstract, and to which the reader is therefore referred. We shall merely remark, that the plan of diagnostic bloodletting, as recommended by Dr. Hall, is even as yet scarcely appreciated, and that when judiciously carried out it will frequently be the means of avoiding error, not only in the case in question, but in the many pseudo-inflammatory diseases which the readiest tact occasionally fails otherwise to diagnosticate.

In connection with the subject of apoplexy, we may mention a paper read a short time since before the Medico-Chirurgical Society by Mr. Hewett upon extravasations of blood within the cavity of the arachnoid. The author distributes these effusions into four principal groups; 1st. Those in which the blood is either liquid or coagulated, in the latter case being spread out in the form of a membranous layer ; 2d. Those in which the extravasation presents itself in the shape of a false membrane ; 3d. Those in which the blood is enveloped in a sac having every appearance of a newly-formed serous membrane ; and 4th. Those in which the blood is fluid and encysted. Of these the third division is important in a physiological sense, as it tends to confirm the opinion that the blood is capable of undergoing organization by an inherent action, quite independent of that of surrounding tissues. In other respects the observations of Mr. Hewett are for the most part in accordance with those of MM. Becquerel, Legendre, Prus, and Rilliet and Barthez, the latter of whom, however, notices meningeal apoplexy only as it occurs in infants and young children. We may remark, *en passant*, that the rare occurrence of true sanguineous apoplexy in an infant only eleven days old, has recently been observed by Dr. Campbell.

7. *Ramollissement*. The latest researches upon the subject of cerebral softenings are those contained in a memoir by M. Rochoux. The opinions of this writer differ from those of many previous authors, as Lallemand, Cruveilhier, Carswell, and more especially Durand-Fardel, in the affirmation that the softening of the cerebral substance, which is so commonly seen in the neighbourhood of apoplectic clots, is the precursor and not the consequence of the hemorrhage. This form of softening to which he applies the term "hæmorrhagiparous," he believes to be present in 99 cases out of 100 of sanguineous apoplexy. This is, doubtless, rigidly considered, too exclusive a view of the case, but we are nevertheless inclined to coincide with the author in so much as this—that although sanguineous effusion may undoubtedly occur in the midst of perfectly healthy cerebral substance, that such an occurrence is exceptional rather than frequent; if it were not so, the proverbially uncertain tenure of life would be rendered still more uncertain, taking into account the many causes of vascular excitement to which the brain is continually subjected. The objection to this view which some pathologists, and Fardel among the number, have advanced, that if this precursory change in the cerebral pulp were so often present, it would more commonly make itself known by symptoms, is, as M. Rochoux justly observes, quite invalid, for how frequently do molecular changes go on unsuspected in organs, the investigation of which is far more easy than that of the brain?

M. Rochoux is likewise at issue with Durand-Fardel and others with respect to the pathological indications of the cavities filled with a yellowish coloured serum, which are frequently found in the brains of persons who have had an apoplectic seizure. He believes them in all cases to be the remains of apoplectic clots; M. Fardel, as may be remembered, considers that they are indicative of softening only, and not of hæmorrhagic effusion. The question, which is one of great interest, must be regarded yet as sub judice, for until we are able to appeal to a numerous series of carefully recorded cases of persons dying at progressively advanced periods after apoplectic seizure, no conclusions can be arrived at which can be received as worthy of confidence. M. Fardel has, we are aware, attempted this to a certain extent, but a more numerous selection of cases is required in order to give that value to his deductions which he is disposed to attribute to them.

The dependence of white softening of the brain upon suspension of a portion of the cerebral circulation, has been long recognized as an occasional consequence of ligature of the carotid artery. In a case which has lately been reported by Dr. Todd, the analogy of this lesion to senile gangrene is very distinctly shown. The patient was the subject of dissecting aneurism of the aorta, which had completely arrested the flow of blood through the right carotid artery.

Soon after the original seizure, which partook of the nature of syncope, he became paralytic on the left side. After death the right centrum ovale exhibited a perfect specimen of white softening, the entire hemisphere being anæmic. The case is one of peculiar interest in other points of view, and will well repay the perusal.

8. *Cerebral Abscess.* The intimate connexion between abscess of the brain and affections generally of strumous origin, commencing in one of the structures of the internal ear, has been made the subject of a communication by Dr. Cormack, which, however, need not detain us, as it contains nothing with which the profession has not long been familiar.

9. *Encephaloid Disease.* Dr. Cowan has published two cases of this rare disease of the brain. As might be supposed, although there was ample evidence, in both instances, of organic mischief in the cerebral mass, nothing transpired to afford the slightest clue, at the time, to the real nature of the disease. Dr. Cowan, however, thinks, that as far as the evidence of these cases can assist the diagnosis, that malignant disease of the brain may be suspected when, in addition to distinct symptoms of organic change, pains of a neuralgic character are present, and are accompanied by gradual emaciation and a cachectic appearance.

10. *Epilepsy.* The only observations worthy of record upon this subject, as involving anything of novelty, are those of M. Selade; who has devised a method of treatment, founded upon the fact of the occasional suspension of the disease by the supervention of intermittent fever. His plan consists in the endeavour to establish an artificial intermittent, by the use of such means as bring about a state resembling the several stages of the genuine disease. Thus, in order to induce the factitious cold stage, the patient is submitted to a prolonged immersion in cold water. He is then placed in a heated room, and covered with bed-clothes, until the hot and sweating stages are counterfeited. Dr. S. states, as the result of his observations, that after the repetition of this process for a few times, at the same hour, the artificial intermittent establishes itself without the intervention of the bath; and details two cases in which obstinate epileptic attacks were thus completely and permanently removed. The subject of epilepsy has also been treated of, if in a less novel, certainly in a less fanciful manner, by M. Rodier, who speaks highly of the scalp issue, and by Dr. Blackmore, whose remarks we have elsewhere given.

11. *Neuralgiæ.* Among the additions to our knowledge in this important class of diseases, we may refer to a monograph by M. Merat, which treats principally of the painful affections of the ganglionic nerves. In the treatment of these, the author recommends the powder of valerian, and the Sedum acre, in large doses. The Cannabis Indica has likewise been suggested as a remedy for neuralgic diseases, by Mr. Donovan; as also tobacco, by Mr. Chippendale; the expressed juice of the mistletoe, by Mr. Hardy; and electricity, by Professors Wisgrill and Wirey. The extract of misletoe is used in the form of a plaster, applied over the affected nerve. The pain is said in most cases to subside in a few minutes. In addition to these instances, we may mention that Mr. Rynd proposes the treatment of obstinate tic by the direct inoculation of the nerve with narcotic substances. He relates two cases, in which the acetate of morphia, in solution, introduced by means of puncture, was eminently successful. In reference to this suggestion, we may remark, that Mr. Rynd has been anticipated by M. Jacques, (*Annuaire de Therapeutique*, 1844,) who appears some time since to have adopted the identical process which he has recommended.

12. *Tetanus.* The periodical literature of the last few months holds out to us considerable encouragement in the treatment of this usually fatal disease, by the record of several cases, in which the means adopted have been followed by success. Of these Mr. Donovan relates two, and Professor Miller one, each of which yielded to the employment of the Cannabis Indica. The cases which occurred to the former writer place the powers of the medicine in a very favourable view; the first patient having recovered after taking 134 grs., although before its exhibition the spasms recurred every four or five minutes. According to Professor Miller there is a marked tolerance of this remedy in tetanic cases, and the unpleasant effects usually consequent upon its prolonged use are seldom observed.

Two cases have likewise been lately recorded in which material benefit, and in one case complete success, followed the use of ardent spirits in intoxicating doses. The first case is one related by Mr. Stapleton, in which the tetanic spasms were entirely suspended, but the patient was not saved. The second occurs in a paper read before the Medico-Chirurgical Society by Dr. Wilson. The patient, who was under the charge of Mr. Ilott, of Bromley, was obviously cured by the exhibition of brandy in enormous quantities, opium being at the same time studiously avoided. During the period of eight days, the patient took as much as two gallons of brandy, in addition to wine, &c. The discussion which followed the reading of this case chiefly turned upon the value of opium in tetanus, upon which the most opposite opinions appeared to be entertained. Among those, however, who supported the relater of the case in his opinion of the inutility of narcotics, were Dr. Wilson, Mr. Solly, and Mr. Curling. The subject of tetanus has also been treated of by Dr. Inglis, and Mr. Stafford. Amid the conflicting testimony which we meet with respecting the treatment of this fearful malady, it is difficult to arrive at any satisfactory decision as to the plan most likely to be successful. The fact, however, that the majority of tetanic patients appear to die by asthenia, affords strong evidence in favour of the propriety of the method adopted in the cases last related.

### § IV.—*Diseases of the Respiratory System.*

Upon this class of diseases we have to notice many interesting communications. Of those which relate to the diseases of the respiratory system in general, we call the attention of our readers with peculiar pleasure to a paper by Mr. Sibson, which has for its object the determination of the topographical changes which the thoracic organs undergo in disease. The value of this laborious essay in the study of chest diseases cannot be too highly estimated ; and whoever fails to make himself familiar with its contents, will be without excuse for those errors in diagnosis which it is so well calculated to obviate. We offer no apology for thus alluding to a work which, though recent, does not strictly come within the prescribed limits of our report, the fact that it is published in a form which precludes its general circulation, is, we conceive, a sufficient reason for its mention.* It is not our intention to analyse this paper at the present time, as we shall have to refer to it on more than one occasion. We shall pass at once to the subject of

13. *Phthisis Pulmonalis.* The first work which we are called upon to notice under this head, is one recently published by Dr. Evans, in which doctrines widely different in many respects from those in common circulation are enunciated, but which will not, we imagine, be very generally received. According to the views of this author, that product, which we have always been taught to consider as of primary importance in the pathology of the disease, namely, tubercle, plays but a secondary part; being no more, to use his own expression, the cause of the disease, than the pus is of pneumonia. Neither are the symptoms of phthisis allowed to depend upon the presence of this deposit; the emaciation, hectic, cough, &c., being all ascribed to pulmonary irritation, and active pulmonary congestion, combined with what he terms a "diminished force of growth." As may be surmised from these opinions, the author is a believer in the inflammatory origin of tubercle; that product being, according to him, only a modification of pus, and secreted under certain states of constitution in the same manner. Pulmonary irritation is, with him, the most important condition to which the consumptive patient is exposed, that being in all cases the precursor and cause of the more serious and fatal symptoms. It is necessary, however, to state, what it is that the author designates by the term irritation. He observes that in a pathological point of view, irritation consists in the presence of a diminished quantity of blood in the affected tissues, which, in consequence, become dense, contracted, and pale, the natural secretions being at the same time suspended. This condition as it occurs in the lung he considers to be declared by an exaggerated respiratory murmur. We must here remark that, although we may not be disposed to object to the author's definition of irritation, if by that term

* Since the above was written we are happy to find that the essay alluded to has been published in a separate form.

he means to designate the same state of capillaries which, as in the experiments upon the web of the frog's foot, (James *on the Nature of Inflammation,*) is brought about by a puncture or other means, and is the precursor of those vascular changes to which the term inflammation is applied, we must still doubt that such a condition ever precedes the deposition of tubercular matter, in the relation of cause. It is, moreover, we must confess, perfectly unintelligible to us how a state of lung in which the "tissues" are "dense," "contracted," and "pale," and in which, therefore, the air-cells must be diminished in capacity can be evidenced by a sign which presupposes the admission of a preternatural quantity of air.

We shall pass over the author's opinions respecting the origin of tubercle, and briefly allude to his mode of treatment. This he divides into sections corresponding to the different stages of the disease. We have, therefore, the treatment of the phthisical predisposition, that of pulmonary irritation, of the period of inflammation, and of suppuration.

The first stage, or that of predisposition, is, according to Dr. Evans, signalized by a "deficient force of growth," causing atrophy of the red tissues, and muscular weakness, emaciation being sometimes an accompanying symptom, at other times an increased deposition of fat. In the latter case, especially if the pulse retains its ordinary frequency, [some might reasonably doubt wherein the phthisical tendency consists in these instances] in addition to the means presently to be mentioned, the author allows wine and fermented liquors. In the former, these must be strictly prohibited, and in their place we are to exhibit such medicines as are capable, according to Dr. Evans, of diminishing the "conducting power of the nerves," such as opium and hydrocyanic acid. In addition to this, nutritious diet, pure air, &c., are indispensable. In meeting the second indication, or that of the treatment of pulmonary irritation, the author insists upon the necessity of distinguishing whether the irritation be primary or secondary. The treatment varies in the two cases. The circumstances which generally give rise to secondary irritation of the lungs are said to be chronic inflammation of the larynx, womb, and kidneys. The treatment, therefore, resolves itself into that most suitable to the disease to which the pulmonary complication is sympathetic, combined, it may be observed, with those means best calculated to allay the secondary irritations themselves. The author avoids the mention of the treatment of the uterine and kidney affections, and confines himself to that of the larynx, which consists in mercury, counter-irritation, leeches, seton in the neck, and painting the part externally with iodine or nitrate of silver. The special treatment of the pulmonary irritation is, inhalation of the vapour of water, with counter-irritants, and small doses of opium and prussic acid.

The author now addresses himself to the management of the third stage of phthisis, that of pulmonary inflammation. Here, as he very properly observes, great difficulty arises, in consequence of the fact that those means which are best calculated to subdue the pulmonary inflammation, viz., depletion, &c., are, unfortunately, the most likely to confirm the tuberculous predisposition. The practical tact of the physician is shown in the manner in which he steers between this Scylla and Charybdis in therapeutics. It is here that Dr. Evans relies upon the treatment by repeated counter-irritation, the advantages of which he appears to have just appreciated in rather a disreputable school it must be allowed,* but which, if he is to be believed, is a striking instance of the fact, that good may sometimes arise out of evil. The plan he adopts is that of stimulating liniments repeatedly applied to the whole chest, and consisting of equal parts of spirit of turpentine and acetic acid. In the treatment of the suppurative stage, as laid down by Dr. Evans, there is nothing to detain us.

We have thus endeavoured to lay before our readers a brief abstract of the general tendency of the author's views; from many of which we, however, think it right to express our dissent, premising at the same time that there is much that is indicative of unusual practical acumen, and calculated to excite reflection upon a disease which we are all too apt to abandon as inevitably beyond the reach of our art.

That phthisis pulmonalis occasionally undergoes a spontaneous cure has long been believed upon the evidence of the cicatrices and cretaceous remains which are sometimes found to exist in the lungs of persons in whom the disease had either never been suspected during life, or whose pulmonary symptoms had entirely subsided. Dr. H. Bennett endeavours to prove that this fortunate termination is far more frequent than is generally suspected, and supports his opinion by statistical data, which may be thus briefly recapitulated:—

| | | | |
|---|---|---|---|
| Of | 73 | cases examined by himself, cicatrices, &c. were found in | 28 |
| | 135 | . . . . M. Boudet, . . . . . | 116 |
| | 100 | . . . . M. Rogée, . . . . . | 51 |
| | 308 | | 195 |

So that, of 308 cases, the appearance alluded to was found in more than one half. It would afford us much gratification to be able to believe that Dr. Bennett had not overrated the frequency of the spontaneous cure of tubercle as thus supposed to be indicated. But this we cannot do. We do not doubt in the least degree the correctness of the data he has produced, but the deductions drawn therefrom do not appear to us to be in all cases trustworthy. That the presence of cretaceous concretions in the upper lobes of the lung are a proof of the previous existence of tubercular deposit may readily be conceded, but it may be thought questionable whether the puckered cicatrices which, by the author's own admission, are often found to exist alone, are in all cases to be referred to the same lesion. The

* Under St. John Long.

pulling-in of the surface of the lung may be produced by other causes than that of the healing of tubercular cavities, as for instance by that peculiar contraction of the pulmonary cellular tissue described by Stokes under the term *cirrhosis* of the lung, and also as Fournet has shown by the mechanical effect alone of pleural adhesions. With deference to Dr. Bennett, we are constrained to believe that such has been the origin of the appearance in some of his own as well as in the cases of Boudet and Rogée.

Dr. Hennis Green has called our attention to certain striking differences which are to be observed in the phthisis of young subjects, as contrasted with the same disease in the adult. The principal distinction is to be found in the fact that tubercular matter is more generally diffused through the lung in the infant, and that its consentaneous deposition in other organs is more frequent. Unlike the disease as it shows itself in the adult, Dr. Green has also observed that the disease is often more developed in the lower and middle lobes than in the upper, the same locality being commonly the site of caverns, when none exist in the upper lobes. This is a practical fact of the great value, and one the knowledge of which may prevent much misapprehension in diagnosis as founded upon the auscultatory phenomena of the disease. The symptoms of phthisis in the child are in many cases exceedingly obscure, since, as has been noticed by Rilliet and Barthez, we are unable to derive the same positive information from the stethoscope as is afforded by it in the adult. The harsh respiration under the clavicle in particular, which is so valuable a sign of tubercular deposition in the latter, may exist to an intense degree in the young child without the slightest lesion of the pulmonary tissue. The cough and expectoration are likewise in the child far from commensurate with the extent of the disease, and hæmoptysis loses the value which the investigations of Louis has attached to it in the adult, as it does not occur oftener than once in five times.

It is also difficult in some instances to distinguish tubercular deposition from lobular pneumonia, for, as has already been stated, the locality of the disease will not, as in grown up persons, afford us any evidence from which the real nature of the consolidation may be presumed. The diagnosis as stated by Rilliet and Barthez, is founded mainly upon the period at which bronchial respiration becomes perceptible, being much earlier in pneumonia than in tubercular infiltration, and less persistent in the former than in the latter. These are, however, it must be allowed, but indifferent rules, and it is to be feared that there are no positive signs by which a sure diagnosis is to be formed. More assistance will be derived in such cases from the history of the attack, and its amenability to remedial measures than from either the stethoscope or rational signs.

We are indebted to Dr. Addison for an excellent essay, in which he points out the important part assumed by inflammation in all cases of tubercular phthisis. He describes the disease under three separate divisions: 1st. Pneumonic Phthisis; 2d. Tuberculo-pneumonic Phthisis; 3d. Tubercular Phthisis. The first division constitutes one form of what in common parlance is termed "galloping consumption," and may be either acute or chronic. Of the acute form the author recognizes three varieties, one in which some attempt at reparation is made, as is shown by the presence of gray induration; a second, is that of inflammation arising about the circumference of old induration, and destroying both it and the newly-invaded structures together; the third variety not mentioned.

Of the chronic pneumonic phthisis he admits two subdivisions; one in which old indurations are converted into vomicæ; another in which the lung is universally invaded by gray induration, but is not disintegrated. We regret that the author should seek further to complicate a subject already sufficiently distant from simplicity, by employing the term phthisis in its etymological sense to a disease which has nothing beyond its fatality in common with that to which the term is limited by common consent. We can see no possible good which can compensate for the confusion which may be produced by bringing under the same denomination the affection above described, which is evidently no more than simple pneumonia, and is strictly unconnected with true tubercular consumption.

Under the title of tuberculo-pneumonic phthisis, is described a common form of the complaint in which, although tubercles are present, and the lungs may even be studded with them, the efficient cause of the fatal termination is pneumonic inflammation. The tubercles in these cases are of what Dr. Addison terms the *sthenic* kind, and show little disposition to soften.

The third form of Tubercular Phthisis is the disease properly so called, and of which tubercles of the *asthenic* kind form the essential element. In these cases excavations arise from the softening of the tubercular matter itself, and independently of the destruction of the pulmonary tissue. The author concludes his communication by an examination of the several forms of thoracic disease which may be mistaken for phthisis. These are chiefly:—1. Recent pneumonic inflammation on the upper lobes of the lung; 2. Various forms of pulmonary induration: 3. Pneumonia in the third stage; 4. Simple bronchitis confined to the upper lobes; 5. Dilated tubes; 6. General or partial pleuritic effusion; 7. Pulmonary apoplexy; 8. Malignant disease of the lung.

*Antagonism of Phthisis and Intermittent Fever.* This remarkable fact first pointed out by M. Boudin has received further confirmation from the observations of MM. Triber, Wolheim, Mayer, Gouzee and Wœmer. M. Fourcault, on the contrary, who appears to have examined this affection in a truly philosophical spirit, has come to the conclusion opposed to M. Boudin, that phthisis and intermittent fever are not mutually exclusive, and that if they sometimes appear isolated, they as often co-exist.

In the treatment of phthisis M. Cossy recommends the exhibition of alkalies, combined with the inhalation of ammoniacal vapours, and M. Forget the recurrence to the ancient remedy, opium. The former, it may be observed, is an unimportant modification of the method proposed some years back by Dr. Campbell, and has been found by Louis equally inefficacious with all other boasted remedies.

14. *Tubercle.* The anatomical site of the deposit is thus stated by various late writers :—

It is considered to be generally deposited in the elastic cellular tissue of the lungs, less frequently in the air vesicles, and capillary bronchial tubes, by Lebert; in every structure which enters into the composition of the lungs, in the coats of the pulmonary artery, in the walls of the bronchial tubes, in the air cells, and upon and under the pleura, by Sibson; in the interior of the pulmonary cells and the minute bronchial tubes, by Nicolucci and Mr. Rainey. Tubercle is considered to be a modified form of albumen by Evans and Bennett; to have no analogy to pus, by Lebert, or to fibrin, by Rainey. According to the observations of the latter writer, tubercle may be distinguished from fibrin by an examination of the neighbouring capillary vessels. Those adjacent to tubercle retain their natural character, those, on the contrary, which are seen in the vicinity of a fibrinous deposit, are tortuous and varicose. These observations, it may be remembered, are to a certain extent at variance with the researches of Guillot and Van der Kolk mentioned by Louis, (*Walshe's Transl.* p. 29,) namely, that the branches of the capillary pulmonary vessels, so far from being natural, stop short, as they approach tubercular deposit, leaving a space of about two lines in breadth, which for a time at least remains perfectly destitute of vascularity.

The inflammatory origin of tubercle is maintained by Bennett, Evans, and Sibson; denied by Nicolucci and Lebert. We have thus, it appears, made no nearer advances to the settlement of the question. The doctrine of the inflammatory origin of tubercle has, however, always appeared to us untenable; for, setting aside the objections arising out of the relative topographical statistics of this product, and those of inflammation, as established by Louis, there are others to be adduced, which it is equally difficult to reconcile with the doctrine. Tubercle undoubtedly exists as such in the blood; it must therefore either coexist with the fibrin, or be supplementary of it. The latter supposition is entirely negatived by the researches of Andral and Gavarret, which demonstrate that the blood of phthisical patients contains more fibrin than that in any other disease, the pure phlegmasiæ excepted. We must, therefore, of necessity, admit their coexistence in the blood. This being granted, the question of the origin of tubercle, as far as inflammation is concerned, is reduced within very narrow limits. Either the same vital action, i. e. inflammation, existing in capillary vessels, which are separated from each other by inappreciable distances, is capable of giving rise, at the same time, to two totally different products, namely, tubercle and fibrin,—or we must conclude that these products originate in a different vascular action. In other words, we must believe, if we hold the inflammatory theory, that the tubercular deposition, and the chronic induration in its immediate vicinity, the latter a pure fibrinous exudation, are the result of the same action in contiguous capillary branches. Now, although we do not maintain that it is impossible, we must yet hold that it is improbable that, when the blood holding both the fibrin and tubercular matter in solution, arrives at the site of two given capillaries—one supplying one cell, the other the next—these vessels should be capable of exercising a kind of elective affinity; and one under inflammation, allow the exudation of tubercle—the other, of the true blood-plasma. The difficulty is only to be avoided upon the supposition that of these two processes, namely, tuberculization and pneumonic exudation, going on, as they assuredly do, at the same time, the latter alone is inflammatory, the other is the product of some other kind of vascular action, most probably that which in healthy subjects is subservient to normal nutrition.

On the subject of the general pathology of tubercle, we may direct attention to a memoir by Dr. Cless, to another by Engel, and to the researches of Lebert, before alluded to, the summary of which will be found in our Abstract.

15. *Asthma.* Mr. Harrison has recommended a trial of the fumes of nitrate of potash, in spasmodic asthma, during the paroxysm. The room which the patient occupies is to be filled with the fumes by burning paper which has been dipped in a saturated solution of the salt. This suggestion is not new, the plan having been some time since adopted in America; it is, however, worthy of attention, from the facility with which its powers may be tested.

16. *Black Pulmonary Matter.* The dark-coloured deposit which is frequently found in the respiratory organs of old persons, has been minutely investigated by M. Guillot, without, however, his having contributed much to the elucidation of the subject. The appearance in question was, it is well known, first described by Laennec, and was by him, as well also as by many subsequent writers, attributed to the inhalation of carbonaceous matters. In the cases which are referred to by the author, this opinion could not be entertained, as the patients had never been exposed to circumstances under which the inhalation of such matters was likely to occur. The theory which is favoured by M. Guillot, is that which was long since enunciated by Dr. Carswell, (*Cyclopæd. Pract. Med.*—Art. *Melanosis,*) namely, that it is due to stagnation of the blood in the pulmonary tissues. This view of the case is supported by the consideration, that the instances in which this discoloration of the lung is found, are exactly those in which we should *a priori* expect the pulmonary circulation to be liable to congestion and stagnation; namely, in the old, and in persons whose lungs are emphysematous. Although this black deposit is a physiological rather than a pathological change, it is capable, when it occurs in great abundance, of producing serious mechanical inconvenience, by obstructing the capillary vessels and respiratory canals. The remarkable property possessed by this matter, as stated by M. Guillot, is its power of inducing a modification in tubercular matter. He believes that it operates as a spontaneous check to the further deposition of that product, by obliterating the new system of capillary vessels, which, he has observed, replaces the obstructed capillaries of the pulmonary artery.

17. *Empyema.* This subject has met with a considerable share of attention of late, more particularly in reference to the operation of paracentesis. Upon the safety and propriety of this operation, the profession is still as much as ever divided; some maintaining the opinion of the late Dr. Hope, that all cases which are curable with, are also to be cured without it; others holding an intermediate opinion, that it is only to be advised as a dernier resort, to avoid impending suffocation; others, again, contending for its positive utility as an early procedure. Among the supporters of the latter view, Dr. Hamilton Roe is pre-eminent, having furnished us with a valuable essay, which will be found in a condensed form in the preceding part of this work and the object of which is to show, by numerical proof, that the operation is both safe and advisable, whenever the fluid either fails to be absorbed, or is clearly ascertained to be purulent. For a more detailed account of the author's practice, in order to avoid repetition, we refer our readers to the abstract, premising that, although we cannot entirely agree with him in the favourable views which he takes of the operation, we must admit that he has placed it before the profession in a far more enticing shape than had been done by any preceding writer.

Among other information connected with the subject of paracentesis or empyema, we may mention a report of four cases operated upon by M. Roushille, three of which were fatal; and of three performed with success by M. Trousseau in the last stage; Dr. Durop also speaks of it in favourable terms, and M. Lutjaerens has reported a case in which a cure was obtained under the most unfavourable circumstances by the injection into the pleura of a weak solution of iodine.

Dr. M'Donnell has remarked the existence of a peculiar crepitation in the lungs after the absorption of pleuritic effusion. The sign he endeavours to explain upon the supposition that it depends upon the entrance of air into the lung which had been compressed, and which from its compression has also become œdematous. This view of the phenomenon has subsequently been supported by Dr. Ojier Ward, having been called in question, as it appears very justly, in the editorial remarks of the Lancet, and there referred to that class of sounds which are known to be produced by the contact of roughened serous surfaces. Whatever be the true theory of the production of this phenomenon, Dr. M'Donnell is not the first to notice it, as it has been long since alluded to by Gendrin, and more recently by M. Damoiseau.—(*Archives Generales de Medecine*, 1843.)

18. *Pneumonia.* On the pathology of this disease the only remarks worthy of record are those of Mr. Sibson, contained in the valuable paper above alluded to, and those of M. Prus. The essential and primary seat of the disease is stated by the former writer to be, the capillary branches of the pulmonary artery and vein, the coats of which vessels become, through a "modification in their cell-life," soft and yielding. He agrees with Stokes in discriminating a stage prior to this, in which the minute crepitating bile is observed, and which is recognized by an increased and hissing vesicular respiration. M. Prus, in the memoir above alluded to, has examined the different theories upon the subject of the primary seat of pneumonia with great minuteness. After criticising the opinions of Laennec, Andral, Lobstein, Grisolle, and other French writers, he pronounces his adherence to the opinion which has been previously somewhat indefinitely stated by Lallemand, that pneumonia is essentially an inflammation of the intervesicular cellular tissue, and does not necessarily involve any lesion of the bronchial tubes or air-cells.

In the treatment of the suppurative stage of pneumonia the iodide of potassium is much praised by Dr. Upshur. We have frequently had occasion in practice to witness the advantages which the author attributes to this remedy, particularly in the pneumonia of infants, as it occurs during the progress of or immediately after measles.

### § IV.—*Diseases of the Circulating System.*

19. *Diseases of the Heart.* The contributions to the study of the diseases of the heart and great vessels during the preceding six months are principally those by Drs. Bellingham, Furnival, and Christison, and MM. Forget and Gendrin. The observations of Dr. Bellingham, which will be found at length in another part of this work, are valuable for the clearness with which the physical signs of valvular disease in particular are laid down. In common with the majority of auscultators, he considers regurgitant diseases of the mitral valve to be indicated by a "bruit" with the first sound, most distinct under the left nipple. In regard to this point we may be allowed to state, that it has long been our opinion, founded upon careful clinical observation, that disease of the mitral valves does not give rise to any bruit whatever, and that, in fact, we have no mean of diagnosing the lesion, excepting by reference to the pulse, which is in itself almost pathognomonic. In looking lately through a list of cases of mitral valve disease, we have been able, within certain limits, (not as extensive as might be wished, it must be allowed,) to gain a numerical confirmation of these views. Of 14 cases of mitral diseases, as ascertained by post-mortem examination, a bruit with the first sound existed in 8, and none in 6. This at first sight might appear to favour the common opinion; but we further find, that out of these 8 cases, another cause capable of regenerating the "bruit," namely, obstructive disease of the aortic valve existed in 6. On the other hand, in the 6 cases of patulous mitral valve in which no bruit was perceptible, neither was there, with one exception, any coexistent disease of the aortic orifice. The exception alluded to, it may also be observed, is not in reality one to which any value can be attached, for the aortic orifice was in that case reduced to a rigid narrow ring, a condition which is generally allowed to be incapable of generating a bruit. We conclude, therefore, as far as so small a number of observations will warrant our coming to any deduction at all, that a patulous condition of the mitral valve does not give rise to a "bruit," but that the sound heard in such cases is due to a co-existing lesion of the aortic orifice.

Dr. Furnival's work is a careful resumé of the ordinarily received doctrines of the day, but adds little to our previous knowledge. He particularly insists upon the advantage of giving alkalies in the treatment of acute rheumatism, as a means of preventing cardiac complication. The formula preferred by him is:—Liq. potassæ ʒss; Vin. colchici ♏ xx; Infus. sennæ, or Aquæ menthæ ℥j three times a day. He likewise speaks highly of aconite as a sedative in heart disease, and considers it in all cases preferable to digitalis.

M. Forget considers that too much value is attached to valvular sounds in the diagnosis of diseases of the heart. He thinks that, in order to arrive at a correct diagnosis, it is necessary to determine the relative frequency of the lesions of the different orifices, and the relations of those lesions to hypertrophy and dilatation of the parietes. The results of the analysis of several hundred cases has shown him, that the most conclusive sign of a contracted aortic orifice, is dilatation, and generally also hypertrophy of the left ventricle. This is indicated by bulging in the præcordia, increased impulse, and bellows-sound along the track of the aorta. This state of the left ventricle implies also passive dilatation of the other three cavities, so that in disease of the aortic orifice the whole heart is enlarged, giving rise to increased dull space in the præcordial region. Contraction of the mitral orifice is followed by dilatation of the three cavities behind it, but the left ventricle remains undilated. In this case there is neither præcordial bulging, nor increased dulness on percussion.

The practical deductions drawn by the author from these views are:—that in aortic stricture, with hypertrophy and dilatation of the left ventricle, debilitants and sedatives may be used without fear; whereas, in cases of mitral stricture, these means must be used with caution, as the left ventricle not being thickened, requires all its energy.

20. *Pericarditis.* The occurrence of this disease as a complication of scarlatina, has already been mentioned (vide p. 3.) Mr. Sibson speaks of a mild form of pericardial inflammation, which he believes to occur some time or other in the life of almost every individual. He is induced to come to this conclusion from finding a small quantity of fluid, and a delicate fibrinous deposit on the auricular appendages in the majority of post-mortem examinations in persons dying of lingering disease of the chest, injuries, &c., which came under this notice.

21. *Aneurism.* The diagnosis of aneurisms of the aorta forms the subject of a comprehensive paper by M. Gendrin, for which we refer to a former part of this work, and is also briefly alluded to by Dr. Furnival.

A peculiar form of dissecting aneurism of the aorta has been described by Dr. M'Donnell, in which the blood had taken a double course, one downwards behind the sigmoid valves, which eventually burst into the pericardium, the other upwards separating the arterial tunics as far as the innominata and subclavian vessels. The symptoms of this lesion are well shown in a similar case which is recorded by Dr. Todd in the 27th vol. of the *Medico-Chirurgical Transactions.* These appear, when the disease occurs suddenly, to be in the first place, a state of syncope, which is evidently due to the sudden abstraction of a large quantity of blood from the general circulation, and its impulsion into the new-formed channel. The tearing away of the cellular tissue connecting the coats of the artery before the column of blood, was in the above case announced by a severe anomalous pain in the course of the arterial trunk.

### § V.—*Chylopoietic System.*

22. *Liver, Cirrhosis.* Dr. Corrigan insists upon the necessity of paying attention to the early symptoms of this disease, as it is in the initiatory stage only that remedial measures can effect any permanent good. The affection is ushered in by repeated attacks resembling "cholic," which are apt to come on after meals, with pain at the top of the shoulder, vomiting, more or less jaundice, and occasionally slimy stools tinged with blood. In the treatment of this condition of things, nothing can be done without total abstinence from ardent spirits and other stimulating liquors, which the patient is prone to indulge in, under the impression that they will relieve the colicy pains by which he is harassed. In addition to this, Dr. Corrigan advises cupping or leeching over the hypochondrium, and the exhibition of mercury until gentle ptyalism is established. When this has been maintained for two or three weeks, we may have recourse with great advantage to this trisnitrate of bismuth.

23. The causes and treatment of *biliary calculi* are thus stated by MM. Duparcque and Dufresne. The causes are the phlegmatic constitution, sedentary habits, too animalized a diet, and the prolonged sojourn of the bile in the gall-bladder. The treatment according to the authors, is to be conducted upon three principles: 1st. To dissolve the concretions; 2d. To facilitate their expulsion; and 3d. To induce such a modification in nutrition as shall prevent their recurrence. In order to fulfil the first indication, alkalies, and especially Vichy water, are the author's favourite remedies. The second indication is attempted by the exhibition of a mixture of two parts of spirit of turpentine and three of ether; or by one of castor oil of sixty parts, ether four parts, and sugar thirty parts, the dose being a teaspoonful every hour. The two remedies which are so much depended upon in this country, namely, opium and the warm bath, are not alluded to. The method of carrying out the third principle of treatment is determined by the manner in which the gall-stones are formed. This, in the belief of the authors, is by the conversion of the fatty matters, and they accordingly recommend abstinence from food rich in oleaginous principles, combined with purgatives, vegetable diet and exercise.

24. That condition of the liver which is familiar to us under the name of *nutmeg liver*, has recently been submitted to microscopical investigation by Vogel. The researches of this observer confirm the ordinary opinion that the appearance is caused by irregular congestion of the organ, and shows that it depends directly upon the contrast exhibited between the pale substance of the hepatic lobules, and the intensely reddened interlobular tissue.

25. A case is described by Dr. Frey under the denomination of inflammation of the vena porta, in which the symptoms were those of phlebitis in general, with the exception that delirium did not occur until the agony of death. This circumstance is accounted for by the author, upon the supposition that the pus did not gain access to the general circulation, being stopped in the portal capillary vessels. The case was fatal, and after death pus was found in the mesenteric, splenic, and in the veins in general which contribute to form the portal circulation.

26. *Abdominal Pulsation.* This is a symptom of very frequent occurrence in practice, and as it is one which never fails to awaken feelings of alarm in the mind of the patient, it will be advantageous to have a clear recollection of the various circumstances under which it may arise. These are well described in an article by Dr. Nottingham, to which the reader is referred. As far as our own experience goes, it is nine times out of ten an unimportant symptom, and is readily subdued by the nitrate of silver in ¼ grain doses, or by the bicarbonate of potash and hydrocyanic acid. The theory of the production of the pulsation is not in all cases very evident, but in those cases in which it is complained of by the patient without being perceptible to the medical attendant, it is probably due to an exalted state of nervous sensibility of the stomach, whereby the pulsations of the subjacent aorta, ordinarily unnoticed, become more or less plainly perceived.

27. *Stomach. Perforating ulcer.* A case of this lesion is reported by Dr. Barlow, which is chiefly worthy of mention in consequence of having given rise to a peculiar condition of parts calculated to throw great obscurity over some of the phenomena of auscultation which are in general the least liable to misconception. The contents of the stomach being extravasated through a circular ulcer, instead of giving rise to a general peritonitis, excited only a circumscribed inflammation, so that a large cyst or abscess was formed communicating freely with the stomach. The contractions of the diaphragm in this case causing the passage of air from the stomach to the adventitious cavity, and vice versâ, gave rise to many of the symptoms of pneumothorax with pulmonary fistula, such as amphoric resonance, metallic tinkling, &c., so that during life the pleural sac was considered to be the part chiefly involved. The case is well reported, and together with the remarks of Dr. Barlow is deserving of attentive perusal.

28. *Intestinal obstruction.* There are few circumstances in which the diagnostic acumen of the physician is more frequently put to the test, than in the discrimination of the exact site of disease in cases of internal obstruction of the bowels. This difficulty may be in some cases materially diminished by the application of certain observations lately published by Dr. Barlow. This author has noticed that a difference is to be found in respect to the urinary secretion, according to the seat of the obstruction. When this is situated in the lower part of the intestinal tube, the renal secretion is little, if at all affected; but when it is higher up, near the duodenum, the secretion has been observed to be more or less completely suppressed. The explanation of this phenomenon is to be found in the fact, that if the obstruction be high up, a small quantity only, or no fluid at all can gain access to the intestines; and absorption is consequently in the same proportion prevented. If the fact be one of general occurrence, Dr. Barlow will have rendered material service to the department of diagnostics, by his notice of it.

The large intestine has several times of late been opened in the lumbar region, as a remedy for insurmountable obstipation. The latest case on record is one by Mr. Evans, of Derby, making the eleventh in which Callisen's operation, as modified by Amussat, has been performed in the adult. This patient recovered from the immediate effect of the operation, but died in consequence of subsequent imprudence.

29. *Dysentery.* A severe form of epidemic dysentery has lately been observed in a union house near Tunbridge Wells, in which the mortality has been as high as one case in four. All methods of treatment appeared to be unsuccessful, until it occurred to Dr. Wilmot to exhibit creosote enemata in the strength of ʒj to ℥xij of starch. Under this plan a rapid amelioration took place.

30. *Peritoneum.* Dr. Spittal mentions a phenomenon attending peritoneal inflammation, which although known as far back as the time of Laennec, has not much attracted the attention of succeeding pathologists. This is a friction sound, analogous to those produced in the pleura and pericardium, and, as in those membranes, depending upon the contact of inflamed serous surfaces. The mechanism by which the peritoneal friction sound is produced is threefold: 1, The respiratory movements and descent of the diaphragm; 2, Pressure on the abdominal parietes by the hand; and 3, The peristaltic action of the intestinal canal. The subject is worthy of deeper investigation than has hitherto been accorded to it, as it is likely to prove a valuable auxiliary source of diagnosis, not only in peritoneal inflammation, but in cases of abdominal tumour.

### § VI.—*Genito-urinary System.*

31. In this branch of pathological study we have to notice the recently published and excellent work of Dr. Golding Bird, the object of which is to supply, in their simplest form, the rules for the discrimination and treatment of the various forms of urinary deposits. As we have, in a former part of this work given an abstract of many of the principal matters contained in the work, we shall merely take a passing survey of its contents in the present place, directing the attention of our readers more especially to the treatment. In the remarks upon the management of the deposits of uric acid and its compounds, we do not find much to arrest our attention, the rules there laid down being, for the most part, such as have long been familiar to the profession. The author, however, introduces one or two observations, which are deserving of further publicity. One of these is in reference to the common practice of exhibiting soda as an antacid. On this subject he cautions us that in many constitutions the continual use of alkaline carbonates is productive of much disorder, and refers to the opinion of Prout, that it occasionally induces the formation of the oxalic diathesis. The other observation to which we allude is with regard to the use of the biborate of soda, which Dr. Bird states cannot be given to females with impunity, as it has been known in two instances to induce abortion.

The most valuable portion of the work is undoubtedly that which treats of the oxalic diathesis; for we have in the chapter devoted to the subject a developement of that particular pathological state such as had not previously been accomplished. This diathesis appears from the investigation of Dr. Bird to be far more common than is supposed, and gives rise to a train of symptoms, which may readily be mistaken for hypochondriasis or spermatorrhœa, with which latter malady it is often associated. Dr. Bird demonstrates, as it appears to us, very satisfactorily, the non-dependence of this diathesis upon the presence of sugar in the system; for neither on the one hand has he found oxalate of lime deposits in diabetic urine, nor sugar on the other in that which contains the oxalate of lime. The origin of the salt he considers to be by the conversion into oxalic acid, by the vital chemistry of the kidney, either of urea, or of the elements which in a state of health would have produced that substance. In the treatment of this state of system, the author speaks very favourably of colchicum: but the principal indications are those of strengthening the digestive powers, and to induce a healthy action of the skin, which are readily accomplished by the mineral acids, the sulphates of zinc and iron, and the shower-bath.

32. *Albuminuria.* The most important notice which this disease has received during the preceding six months is to be found in the admirable clinical lectures of Dr. Corrigan, who considers the pathological condition of the kidney to be analogous to that which is known as it occurs in the liver, by the term *cirrhosis.* The first stage of the disease consists in a state of hypertrophy, caused by the deposit of lymph, not as has been supposed, in the tubular structure of the kidney, but in the intervening cellular tissue; the second stage, as in the liver, is marked by contraction. These two conditions, according to Dr. Corrigan, are widely different as to curability: the latter is perfectly irremediable; the former may be removed by judicious treatment. The symptoms are also, he observed, sufficiently distinctive to enable us to recognize the two stages. In the hypertrophied kidney "the urine is abundant, sometimes tinged with blood, albuminous, specific gravity but little altered, with dry skin and pains in the loins. If in addition to this the specific gravity is low, 1.010, and albumen is still present, there is little doubt that contraction has set in, and the prognosis is correspondingly unfavourable.

M. Fourcault, in a work recently published upon the causes of chronic diseases in general, gives the following rationale of the occurrence of albuminuria. He had long observed that the presence of albumen in the urine, in by far the majority of cases, coincided with notable derangement of the functions of the skin. Struck with this circumstance, he had recourse to experiment to determine whether the two events had any necessary connection. The result was, that in artificially suppressing the cutaneous transpiration, he induced albuminous urine. The explanation of the fact is founded upon the hypothesis that the albumen of the blood is retained in a fluid condition by a chemical union with soda, which alkali is neutralized by the lactic acid retained in the circulation in consequence of its non-elimination by the skin. The albumen thus isolated is rendered as it were an effete element, and is therefore, discharged by the kidney as the most active emunctory of the body. The feasibility of this theory is, according to the author, supported by further experiments, in which he injected lactic acid into the veins of different animals, with the constant effect of causing the appearance of albumen in the urine. These observations, which are for the most part in accordance with the views of Mr. Ross upon the same subject, are deserving of close attention; for they, if true, entirely subvert the opinion of the illustrious physician whose name has by common consent been accorded to the disease for the elucidation of which he has done so much. In fact, if the skin have in reality so great an influence upon the appearance of albumen in the urine, we must undoubtedly cease as hitherto to regard the congestive or inflammatory lesion of the kidney as of primary consequence in the causation of the disease. We, however, look upon this branch of pathological inquiry as far from being satisfactorily determined. We know thus much, that there exists a train of symptoms indicating a generally fatal combination of pathological conditions, but of the three main elements of the disease in question —the presence of albumen in the urine, the appearance of dropsical effusions, and the concomitant lesions of the kidney: we are not able, as it appears to us, as yet to determine the respective place in the series of morbid actions.

The treatment of albuminuria will be found to be well described by Dr. Williams. In the early stage of the disease in which there is usually more or less pain in the back and loins, local abstraction of blood by cupping will be found of material benefit. The dropsical symptoms may be attacked by hydragogue catharies, diaphoretics, &c. Diuretics are, according to Dr. Williams, unsafe until the renal congestion has been in a measure removed. In the chronic form of the disease, Rayer recommends small doses of tincture of cantharides, and Mr. Kidd, the establishment of an issue in the loins. We regret that we do not find more specific notice of the treatment of some of the more urgent symptoms attendant upon that fatal malady ; as, for instance, of the vomiting which in many cases forms so insurmountable an obstacle to the administration of medicines, and by the rejection of all food tends mainly to the fatal determination. On the subject of treatment of albuminuria as it occurs after the eruptive diseases, we have already had occasion to remark in a former part of this Report.

§ VII.—*Diseases of Uncertain Seat.*

33. *Scrofula.* The most recent, and at the same time the most important communications with respect to this common source of the deterioration of the human species, are to be found in the late work by M. Lugol, of which we have had the honour of producing an English translation. The object of this work is strictly confined to the investigation of the causes of the disease, the author having, as he conceives, sufficiently exhibited his method of treatment in former publications. The main peculiarity in the author's views consists in the great development which he has given to hereditary influences in the causation of the disease, and the small share which he believes external agents to posses, when hereditary taint is not also present. No combination of circumstances in fact can, according to him, make a healthy man scrofulous. These views are unquestionably startling, from the implicit faith with which we have been accustomed to receive the doctrine of the influence of impure air, and bad food, in the production of scrofula; and it is to be regretted assuredly that the author had not followed the philosophical Louis in the mode in which he has related the results of his investigation. Now, although it is not our intention to defend M. Lugol from the accusation of inexactitude with which many of his dicta have been received, we must be allowed to suggest that assertions coming from the lip of a man who, like Lugol, writes a book not to get a name, but at the end of a thirty years' experience, and on the point of retiring from the profession, might be received with a less amount of the support afforded by figures than under other circumstances we should require. The author, among other points, in which he differs from preconceived opinions, states as his experience, that scrofula is most frequently shown in persons with dark hair and complexion. "Scrofula," he observes, "rarely shows itself in persons of light hair and complexion; more than half are dark, and among the remainder the hair is generally of various shades of auburn." As far as our own observations have gone, and we have, since our attention has been so directed, made many inquiries in reference to this particular point, they coincide with those of M. Lugol.

The circumstances under which scrofula may be inherited are, as M. Lugol observes, more numerous than is commonly known. Not only may an individual actually labouring under scrofula at the time of begetting offspring, transmit to them his cachetic constitution, but he may do so if he at any time has been scrofulous, even although he should be apparently cured. It appears, also, that even if he have not exhibited signs of scrofula in his own person, his children are nevertheless not exempt, if his brothers or sisters suffer from the disease; for M. Lugol regards it as an axiom, that if one member of a family be scrofulous, the others are so also in a greater or less degree. Phthisical parents may beget scrofulous children; as may also those who are labouring under secondary syphilis; those who marry too early or too late; who are disproportioned in age, or in the physical vigour of the sex; or who have committed venereal excesses. Lastly, scrofula may be inherited from maniacal, epileptic, and paralytic parents. In point of fact, any circumstance which gives rise to debility in the reproductive system in the parent, may become a source of scrofula in the child.

Our space will not allow of a minute analysis of M. Lugol's important work; we shall therefore pass on to a subject which is nearly allied to scrofula, namely :—

34. *Cretinism.* The pathological history of the degenerate races inhabiting the humid valleys of the Alps, has lately been extended by the labours both of British and continental writers; among which, we shall mention those contained in an interesting brochure by Dr. Wells. The first part of this work is occupied by a physiological discussion upon the origin of the term *cretin*, and by an account of the authors who have written upon the subject. He then proceeds to the description of a cretin, and to the consideration of the proximate cause of the degeneration. It appears, according to Dr. Wells, that cretinism is not hereditary, but that it shows itself usually for the first time about the period of dentition. In this view, however, he is not at variance with Dr. Rosch, who has likewise minutely investigated the disease. The proximate cause is stated to be "an imperfect developement of the individual, dependent upon the condition of the blood, which is deficient both in quantity and quality." The author combats the opinion that cretinism and goitre arise from any peculiarity in the Alpine waters, but agrees with Dr. Rosch, in attributing the condition entirely to the injurious operation of the warm damp atmosphere which stagnates at the bottom of the Alpine valleys. The subject has also been noticed in a paper published in the *Transactions of the Vienna Medical Association*, by Dr. Knolz, and in a work by Dr. Troxter.

35. *Rheumatism.* M. Legroux has for some time abandoned bleeding in the treatment of acute rheumatism, and has relied entirely upon the sulphate of quinine. The effects of this medicine are stated by him to be threefold: 1, primary or local; 2, physiological or secondary; 3, therapeutic. The latter only need here occupy our attention. The action of the circulating system seems especially modified by the medicine, the heart's pulsations diminishing rapidly both in force and frequency. The skin at the same time cools down to its natural temperature. The articular affections do not undergo any notable amendment before the third or fourth day, but after that day they rapidly progress towards recovery. The following is a statistical statement of M. Legroux's experience :—Of 24 cases, some had been ill two or three days, others for six or seven, some as long as a month; the mean duration of the illness at the commencement of the treatment was ten days. The duration of the treatment in the successful cases, was 3 days in two, 4 days in six, 6 days in four, from 8 to 9 in four, from 12 to fifteen in two; the mean duration, therefore, was 6 days. Of the twenty-four patients, nineteen were completely cured; in fact, this change from disease to health was almost sudden; there appeared to be no intermediate state of convalescence. The other five had a relapse, but were eventually cured by the same means.

Of these 24 cases there was heart complication in 7; but this did not appear to be influenced in any manner by the quinine.

The preceding facts and considerations lead M. Legroux to the following conclusions:—

1. The sulphate of quinine is a powerful sedative of the circulation.

2. It exercises a powerful influence over the duration and progress of articular rheumatism; it diminishes the symptoms; and probably tends more than any other medicine to prevent the intercurrence of cardiac disease.

3. Given in small and divided doses, it is free from all danger and inconvenience.

4. It often succeeds alone; but it is often useful to associate with it one or two bleedings.

36. *Diabetes.* Dr. Watts has reiterated his opinion, that the proximate cause of this disease is to be sought in the assimilating organs, and that the kidneys are not essentially implicated in the disease, merely acting as emunctories for the sugar, with which, by a vice in the digestive organs, the blood is surcharged. He shows, on the authority of Liebig, that certain articles, such as sugar, starch, gum, &c., in a healthy stomach, are converted into oleaginous secondary principles, which, like the saccharine, are destitute of nitrogen. A further change is then effected by their being converted into animalized, that is, azotized principles, such as are necessary for the constitution of the various tissues of the body. It, however, happens, under certain forms of disease, that digestion is interrupted in one or other of these stages of assimilation, and effects are produced, which vary according to the particular stage at which the suspension of assimilation takes place. In the case of non-nitrogenized principles, if the assimilation has ceased after the conversion of the saccharine into the oleaginous principles, this imperfectly assimilated aliment is, according to Dr. Watts, deposited in the form of fat. Hence, with him, the production of great obesity is one of the precursors of diabetes. If, however, the digestion has not proceeded further than the conversion of the amylaceous matters into the saccharine principle, this remains as an effete matter, and is eliminated by the kidneys and in the alvine discharges. The peculiarity of the views of Dr. Watts, then, consists in his regarding diabetes as made up of three stages: the first, which is essential, and consists in inflammatory gastric dyspepsia, as a consequence of which, the conversion of the non-azotized into the azotized principle is incomplete; the second, which is not essential, and is therefore occasionally absent, in which, from the mal-assimilation of the oleaginous principles, these are deposited in the form of fat; and thirdly, that which is generally recognized as diabetes, in which the digestive process gives rise to a low form of sugar, but is incapable of accomplishing its further assimilation. This supposed connexion of obesity with subsequent appearance of diabetic symptoms, is worthy of further investigation, but we are not aware that it has been insisted upon by any other writer.

In the treatment of diabetes we refer with gratification to a paper by Dr. Imray, in which the influence of warm climates upon the disease is shown to be of paramount importance. A subsequent article likewise contains the report of a case supposed to have been cured by the Peruvian balsam.

37. *Action of Medicine. Narcotics.* Dr. Pickford, of Heidelberg, has made the modus operandi of narcotic medicines the subject of a valuable inaugural dissertation. His conclusions may be thus briefly recapitulated:—

1. A narcotic becomes active only when it enters the circulation.

2. It enters the circulation through the medium of the veins, and not by the lymphatics.

3. Since it is impossible to detect the narcotic either in the blood, or in any component part of the body, it must be allowed to have a special effect upon the nervous mass, the blood being simply the vehicle of the poison.

4. The action of a narcotic differs sensibly, accordingly as it acts directly upon the nervous centres, or only upon a particular nerve.

5. A powerful dose of narcotic poison destroys life by a direct action upon the nervous centres; in smaller doses it kills by its action upon certain nerves, as those of the heart.

6. The nearer the nervous centres that a narcotic poison enters the circulation, the more rapid the death.

*Diuretics.* In the explanation of the modus operandi of diuretics, Dr. Bird alludes to the two following laws:—1st. That substance intended to reach the kidneys must either be in solution, or be readily soluble in the fluids contained in the stomach. 2d. That the solution of these substances must be so diluted as to be of considerable less specific gravity than the liquor sanguinous or serum, i. e. less than 1.028. This strikes us as an important law, and one which is not sufficiently attended to in extemporaneous prescriptions. It readily explains why two drachms of the acetate of potash in a given quantity of water will excite diuresis, while half an ounce in the same quantity will purge. The author has made some remarks upon the laws which are specially worthy of recollection. After alluding to the course which diuretic medicines must take before they reach the kidneys, viz., through the liver and ascending cava to the heart, and thence through the lungs back to the heart, and through the aorta, he observes that when an obstruction exists in any part of this course, a diminished supply of water reaches the kidney. For example, a patient labours under a contracted condition of one of the auriculo-ventricular openings, and dropsical effusions ensue, or he has a contracted liver, and the portal system is consequently obstructed. In cases of this kind no good can arise from goading the kidneys by diuretics, unless the obstruction can first be remedied. This is a point not sufficiently reflected upon in practice, or we should not see stimulating diuretics, as cantharides and squills, so promiscuously given in dropsy, without reference to the condition of the heart and liver. Dr. Bird concludes with these practical suggestions:—

1. Whenever it is desirable to impregnate the urine with a salt, or to excite diuresis by a saline combination, it must be exhibited in solution so diluted as to contain less than five per cent. of the remedy, or not more than 25 grains in an ordinary draught. The absorption of the medicine may be insured by a copious draught of water or other diluent, immediately after each dose.

2. When the urine contains purpurine, or other evidence of portal obstruction exists, the diuretics employed should be preceded or accompanied by mild mercurials, taraxacum or other cholitic remedies.

3. In cases of valvular disease of the heart, it is next to useless to endeavour to excite diuretic action by remedies intended to be excreted by the kidneys. The best diuretics will be found in whatever tends to diminish the congested state of the vascular system, and to moderate the action of the heart; as digitalis, colchicum, and other sedatives, with mild mercurials.

38. *New Remedies.* Some few new medicines have lately been introduced to the profession which we shall briefly notice. The principal of these are the arseniate and the valerianate of quinine and bebeerine.

The *Arseniate of quinine* has been introduced by M. Bourrieres, and is intended by him to be a substitute for arsenious acid, in cases in which that poison is exhibited as an anti-periodic.

The *Valerianate of quinine* has been tested by M. Devay at the Hôtel-Dieu. Its virtues, as ascertained by him, are said to be considerable in those cases in which a combined sedative and tonic effect is required. It is, therefore, serviceable in low forms of fever with nervous excitability; in intermittents of bad character, and in neuralgic and hysteric complaints.

*Bebeerine* is a name applied to a salt extracted from the *Noctandra Rodiei*, nat. or. Lauraceæ, which has been found by Dr. Legan to possess anti-periodic properties of a high order, and is stated by him to be only half the expense of quinine. He records the experience of Dr. Watt of Demerara, and Dr. Nicolson of Madras, both of whom have exhibited it in intermittent fever and neuralgic diseases of various intensities, and who concur in the statement that it is certainly free from those unpleasant consequences, as headache, deafness, &c., which occasionally supervene upon the use of quinine.—*Half-Yearly Abstract of the Medical Sciences.*

